# CovCopCan: An efficient tool to detect Copy Number Variation from amplicon sequencing data in inherited diseases and cancer

**DOI:** 10.1371/journal.pcbi.1007503

**Published:** 2020-02-12

**Authors:** Paco Derouault, Jasmine Chauzeix, David Rizzo, Federica Miressi, Corinne Magdelaine, Sylvie Bourthoumieu, Karine Durand, Hélène Dzugan, Jean Feuillard, Franck Sturtz, Stéphane Mérillou, Anne-Sophie Lia

**Affiliations:** 1 CHU Limoges, UF de Bioinformatique, Limoges France; 2 CHU Limoges, Service Hématologie Biologique, Limoges, France; 3 Univ. Limoges, UMR CNRS 7276 CRIBL, Limoges, France; 4 Univ. Limoges, MMNP, EA 6309, Limoges, France; 5 CHU Limoges, Service Biochimie et Génétique Moléculaire, Limoges France; 6 CHU Limoges, Service de Cytogénétique, Limoges, France; 7 CHU Limoges, Service Anatomie Pathologie, Limoges, France; 8 Univ. Limoges, EA CAPTur, Limoges, France; 9 Univ. Limoges, UMR-7252X-LIM, Limoges, France; University of Technology Sydney, AUSTRALIA

## Abstract

Molecular diagnosis is an essential step of patient care. An increasing number of Copy Number Variations (CNVs) have been identified that are involved in inherited and somatic diseases. However, there are few existing tools to identify them among amplicon sequencing data generated by Next Generation Sequencing (NGS). We present here a new tool, CovCopCan, that allows the rapid and easy detection of CNVs in inherited diseases, as well as somatic data of patients with cancer, even with a low ratio of cancer cells to healthy cells. This tool could be very useful for molecular geneticists to rapidly identify CNVs in an interactive and user-friendly way.

This is a *PLOS Computational Biology* Software paper.

## Introduction

Identifying mutations responsible for inherited or somatic diseases can be essential to define the appropriate therapy for the efficient treatment of patients. For example, this is true for patients presenting an amyloid neuropathy due to Transthyretin (*TTR)* point mutations, who can benefit from new treatments, such as Tafamidis [1]. This is also true for cancer, for which it is important to rapidly detect certain Copy Number Variations (CNVs), such as the 17p deletion, a recurrent abnormality in Chronic Lymphocytic Leukemia (CLL), with major therapeutic implications. Because this acquired chromosomal abnormality directly impairs the *TP53* gene [2, 3], it is now recommended to test this CNV before each treatment for CLL [4]. Indeed, TP53 alterations in CLL are responsible for primary resistance to fludarabine and survival of such patients is clearly improved by new-targeted therapies, such as ibrutinib [5, 6].

High-throughput sequencing techniques allow partial or total sequencing of a patient’s genome. Amplicon sequencing is one of the techniques that enables the sequencing of several thousand exons at a very low cost. Although this method is robust for the discovery of small genetic mutations, such as single-nucleotide polymorphisms or short indels, only a few tools are available for the detection of larger variations, such as deletions or duplications in amplicon sequencing data. Some of these tools require control samples to establish a reference set of data (ONCOCNV [7]). For others (ExomeDepth [8], IonCopy [9], DeviCNV [10], Cov’Cop [11]), control samples are not necessary. Indeed, if the CNV is rare, the other patient samples tested in the same run can serve as controls. In this strategy, multiple patients are tested at the same time, potentially shortening the time to diagnosis.

Most available tools based on the read depth method to detect CNVs include robust statistical methods. ExomeCopy [12] proposes a hidden Markov model to detect CNVs from raw read count data. CONVector [13] was built on a machine-learning algorithm to associate PCR-efficiency correlations for subsets of amplicons. Here, we propose a new tool, CovCopCan, based on the initial read-depth method developed in Cov’Cop, with additional statistical methods and features that allow the rapid and easily detection of CNVs in inherited diseases, as well as somatic data of patients with cancer, even with a low ratio of cancer cells to healthy cells (data sets described in S1 File). CovCopCan includes heuristic methods to compare the value of each amplicon of a patient to those of other patients sequenced in the same run. CovCopCan focuses on data manipulation and results exploration for the interpretation of CNVs. Users have access to an overview of the results for each patient through an interactive visualization, allowing, for example, the exclusion of low-quality amplification from the analysis and quickly restarting CNV detection. In addition, several statistics methods (Loess regression, Cumulative summary) can help in the interpretation of the results.

## Design and implementation

### CNV-detection algorithm

#### Z-score-based CNV detection: “Z-detection”

From the raw read count of each amplicon, CovCopCan applies the same corrections and normalization as the Cov’Cop tool [11], resulting in a normalized read count value (NRC) for each amplicon (see S1 File). Starting from this point, we developed a new CNV-detection algorithm, based on the z-score. The z-score is calculated for each amplicon in each patient, according to the following formula:

$${z-score}_{p\_i}=\frac{{NRC}_{p\_i}-\mu_{p}}{\sigma_{p}}$$

*NRC*_*p*_*i*_ is the normalized read count of the amplicon *i* in the patient *p*, *μ*_*p*_ the NRC average of the patient *p*, and *σ* corresponds to the standard deviation of the patient *p*. The z-score follows a standard normal distribution *N*(0;1). We fixed a threshold corresponding to a significance level of 0.01 for both deletion and duplication events by a one-tailed test. Thus, a negative z-score with a p-value < 0.01 indicates a deleted amplicon, whereas a positive z-score with a p-value < 0.01 indicates a duplicated amplicon. This algorithm automatically determines the best deletion and duplication thresholds based on the variability of a patient's data. The users are free to determine the minimum number of concurrent amplicons required to call a CNV. No minimum distance between amplicons is required, but they have to be on the same chromosome. By default, a minimum of three successive amplicons on the same chromosome was used for all data in this paper.

#### Two-stage ratio to optimize CNV detection

The last normalization step of CovCopCan results in a ratio of standardized patient values that gives a theoretical value of 1 for a gene present in two copies, 0.5 for a deletion event, and 1.5 for a duplication. In this last step, each amplicon value is divided by the median of the same amplicon from the other samples. Once this first ratio is calculated and the first round of CNV detection is performed, a second ratio is calculated excluding all amplicons located inside the initially detected CNVs from each sample, and final CNV detection is achieved. This approach is used to improve standardization in regions in which the same CNV event is present in many patients.

#### Merging CNVs

We provide a “merge” option to reduce the impact of false-negative amplicons on CNV detection. If two CNV areas located on the same chromosome are disjointed by only one amplicon with a z-score duplicated or deleted at a significance level of 0.05, CovCopCan will then merge the two CNV areas to easily highlight this global CNV. In addition, the user can also define the maximum distance value between two CNVs to be merged.

#### Reference amplicon selection or exclusion

For the normalization step, CovCopCan selects a set of amplicons, consisting of those that are the most stable among the patients of a run. These amplicons are then used to normalize the values of the other amplicons. The user can indicate specific amplicons to use for this normalization step (see S1 File). Inversely, our tool also provides the possibility to manually exclude some amplicon data for the last ratio step of normalizations (see S1 File).

#### Control samples

Although CovCopCan works without control samples, it is possible to exploit the presence of controls if they are available. In such a case, the median of the last standardization step is no longer calculated using all the samples but only the controls. Then for each patient, the amplicon values are divided by the median calculated for the controls, according to the following formula:

$${Ratio}_{i\_patj}=\frac{{NRC}_{i_{patj}}}{Md({NRC}_{i_{controls}})}$$

${NRC}_{i_{patj}}$ is the normalized read count of the amplicon *i* in the patient *j*.

$Md({NRC}_{i_{controls}})$ is the median of the normalized read count of the control samples.

CovCopCan can be run with only one control sample but more control samples will improve the result.

### 2D interactive visualization

**Fig 1:**
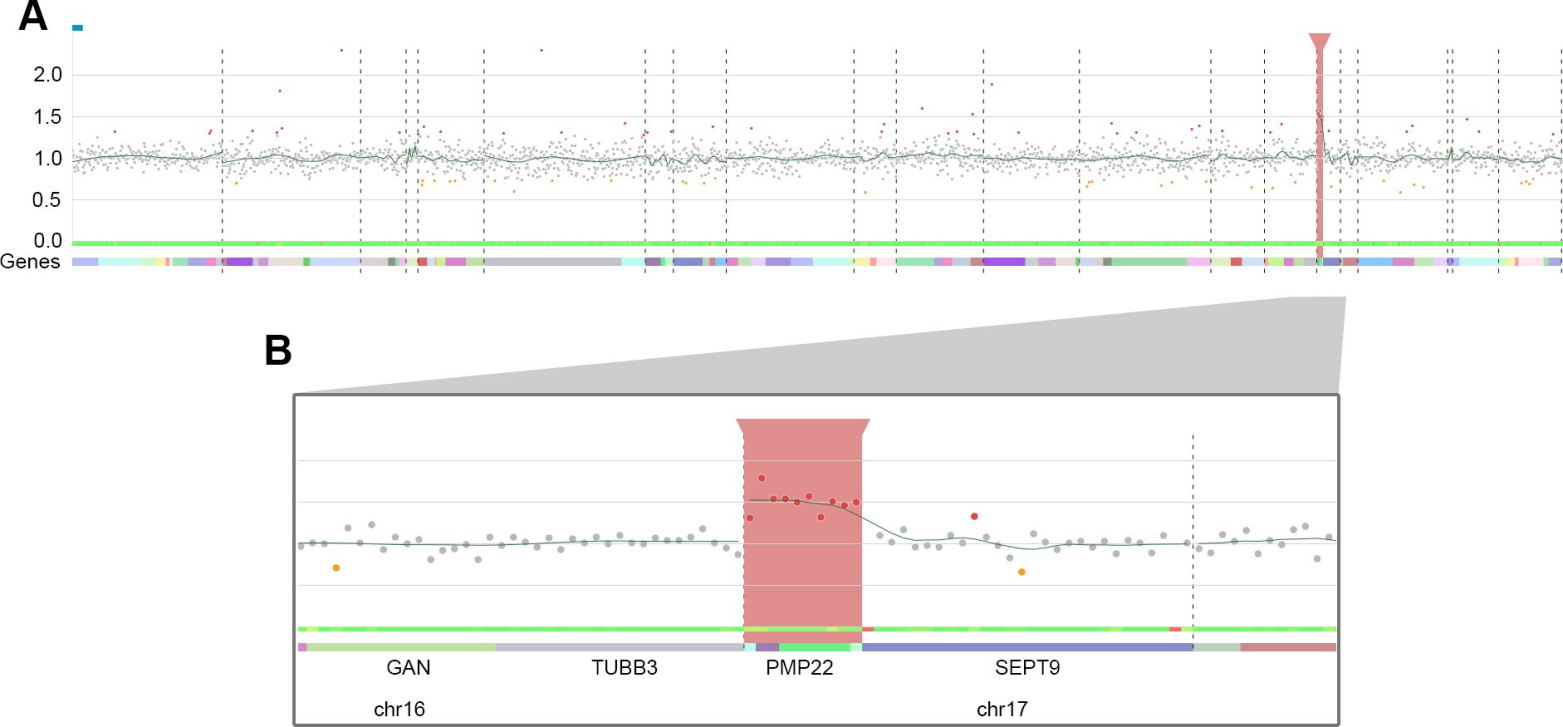
Visualization of CovCopCan. A. General view. Each dot corresponds to an amplicon. The amplicons are distributed on the x-axis according to their genomic position. The y-axis corresponds to the normalized values. Grey dots indicate a “normal” value, whereas red or orange dots indicate duplicated and deleted amplicons, respectively. The names of the gene and chromosome number are located at the bottom of the figure. The green curve shows the Loess regression. The thick green ribbon is a noise heatmap in which green indicates a stable amplicon in all samples (see S1 File). The red rectangle highlights a CNV region. B. Zoom on the duplicated region covered by 10 amplicons (*PMP22*).

An interactive 2D visualization is available for each patient (Fig 1). The amplicons are represented by dots over their chromosomal positions on the x-axis and their normalized values on the y-axis. Users can interactively zoom in on specific regions and navigate between data in an intuitive and interactive way, allowing simple navigation. Several types of information described below have also been added to the graphical representation.

#### Local regression curve

We introduced the possibility to display regression curves on the presented chart to optimize visual CNV detection. We chose to implement the Loess local regression algorithm [14] to easily visualize a sudden change. The Loess regression is calculated for each chromosome. By default, the bandwidth parameter is fixed to 0.25, but it is possible to interactively fine tune it to more or less smoothen the curve. The Loess regression is represented by a green curve on the chart (see S1 File).

#### CUSUM charts

For data generated from cancer or mosaic samples, a sample may simultaneously contain “normal” and deleted/duplicated cells. The deletion/duplication detection accuracy depends on the proportion of deleted/duplicated cells relative to that of the normal cells and the normalized values can be close to 1. CNVs will then be very difficult to detect. Consequently, we added a visual method called CUmulative SUMmary control chart (CUSUM; [15]) to be able to observe a slight increase or decrease in values. For each chromosome, this algorithm calculates the cumulative sum of the positive deviations (values > patient’s average) for deletions and negative deviations (values < patient’s average) for duplications. It can be useful for detecting a slight deviation of the values due to cancer data or mosaicism, as well as small CNVs in inherited diseases.

$$S_{n}^{+}=\max\left(0,\;S_{n-1}^{+}+x_{n}-(\bar{x}+\sigma )\right)$$

$$S_{n}^{-}=min(0,\;S_{n-1}^{-}+x_{n}-(\bar{x}-\sigma ))$$

Here, *x*_*n*_ corresponds to the value of one amplicon, $\bar{x}$ is the mean value of all the patient’s amplicons, and *σ* is the standard deviation. In the visualization of CovCopCan, a blue shape indicates a possible deletion, whereas a pink shape indicates a potential duplication. Although this method makes it possible to highlight potential CNVs, it does not allow precise definition of their breakpoints (see S1 File).

## Results

### Two-stage ratio

**Fig 2:**
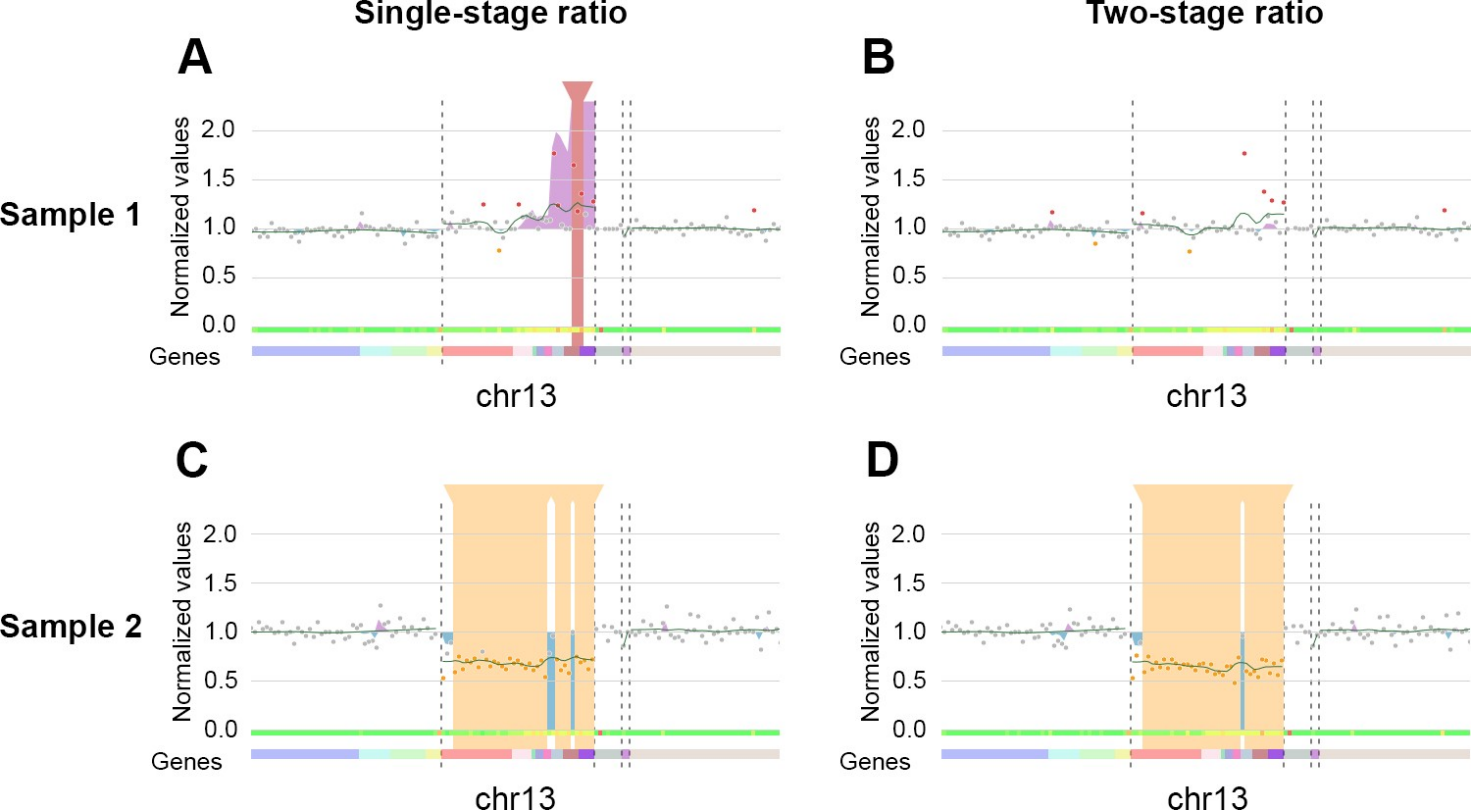
Comparison of single-stage and two-stage ratio results. A. Without the two-stage ratio, a disturbed region showed a false-positive duplication on chromosome 13 covered by three amplicons. B. The two-stage ratio improved the stability of the region and the false duplication was no longer detected. C. Without the two-stage ratio, six amplicons (grey dots in Chr13 area) were not detected as deleted throughout chromosome 13 (39 amplicons) and three separated CNVs were detected. D. With the two-stage ratio, only three false-negative amplicons (grey dots in chr13 area) were present among the 39 amplicons of chromosome 13 and only one amplicon split the total deletion of the chromosome (partial screenshots from CovCopCan).

We visualized the result of the two-stage ratio using sequencing data from panel 2 (see S1 File for details). This gene panel, designed by Ion AmpliSeq designer software, includes 1,206 amplicons on 70 genes. The run presented here was performed on an Ion Proton device and included seven patients. A deletion on chromosome 13 was shared by three of the seven patients (verified by karyotyping). Examples of the visualization obtained for two of the patients (patient 1 normal and patient 2 “deleted”) are presented in Fig 2. Without the two-stage ratio, the region in non-deleted patients was disturbed and a false positive duplication event was detected by CovCopCan in both (highlighted by a vertical red rectangle, as for patient 1, Fig 2A). The two-stage ratio improved the stability of the values so that no false duplication event was detected by CovCopCan, thus increasing the specificity (Fig 2, compare A and B). This method also improved the detection of deletions (highlighted by a vertical orange rectangle) in the true deleted patients, decreasing the number of false-negative amplicons (Fig 2C and 2D).

### Merging CNVs

**Fig 3:**
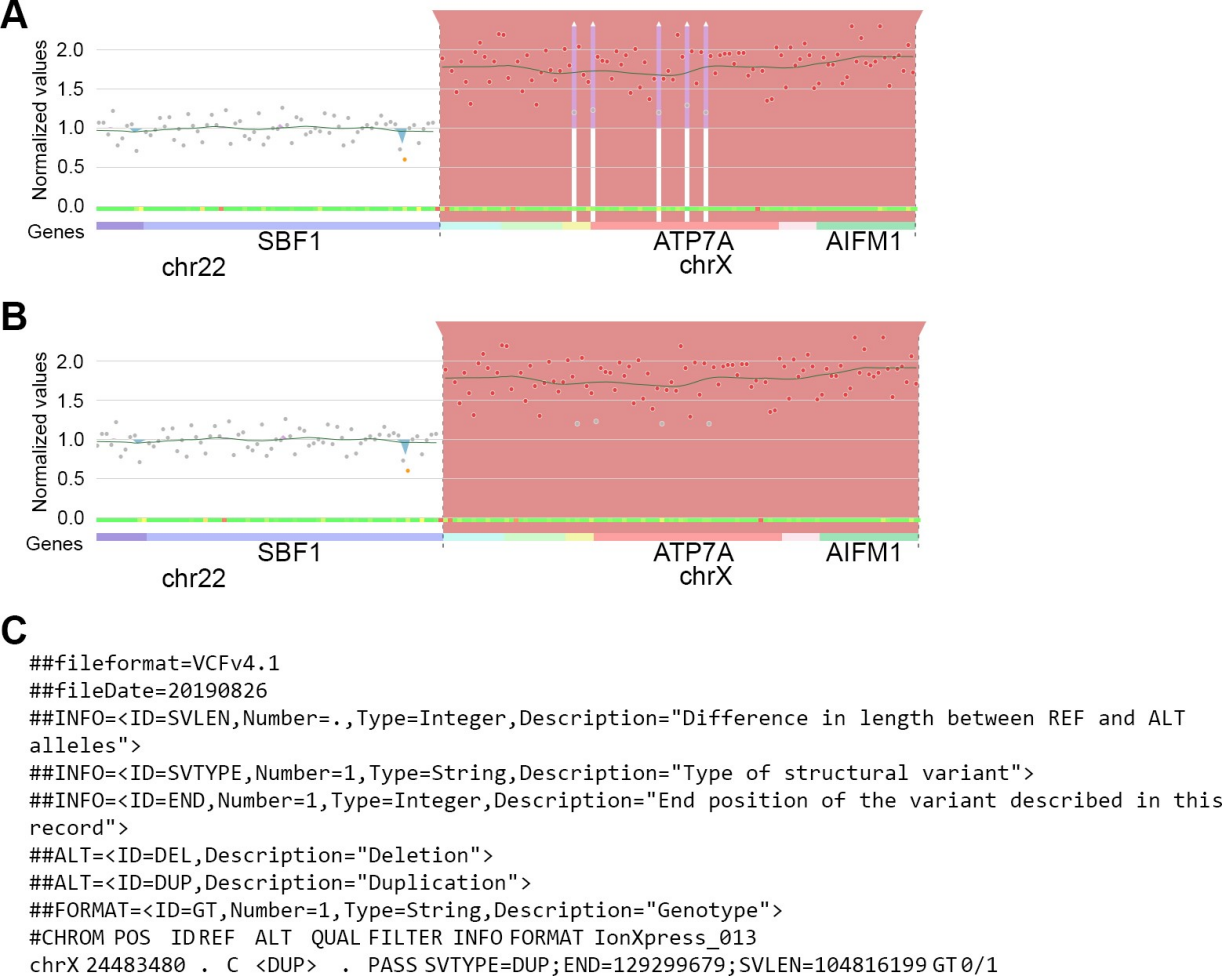
Example of CNV merging on a chromosome X duplication. A. Entire duplication of chromosome X. CovCopCan detects six CNV areas without the merging CNV algorithm. B. By using the merging CNV algorithm, the duplication detected includes all of chromosome X, although some amplicons appear as neutral (grey dots). C. The exported CNV in the VCF format contains only one line corresponding to the duplication of chromosome X (partial screenshots from CovCopCan).

To reduce the effect of individual false negative amplicons, CovCopCan relaxes the significance threshold when a single non-significant amplicon is flanked on both sides by significant amplicons. For this specific amplicon, the threshold will be automatically switched to 0.05. If this amplicon becomes significantly duplicated, it will be merged with the initial duplicated detected areas. The grey dot in the graph will stay grey, indicating that it is a merged area. Deletions are treated the same way. Here, we show the results of this merging option on a complete chromosome X duplication. A single duplication covering the entire gene is detected by CovCopCan, whereas six successive duplications would have been found without this merging option (Fig 3).

### Control samples

**Fig 4:**
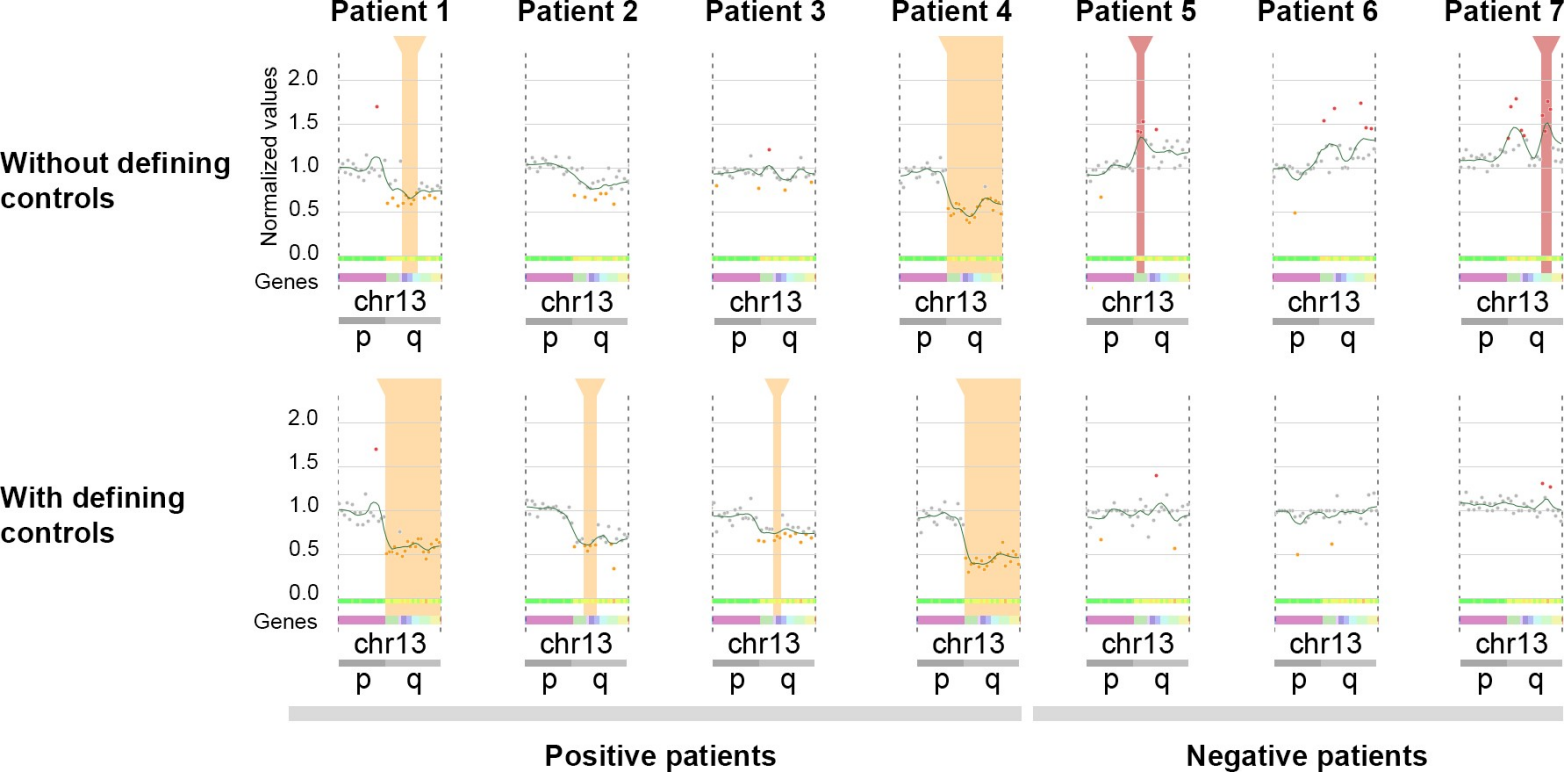
Visualization of chromosome 13 in seven samples. Each dot corresponds to an amplicon. Orange and red rectangles correspond to deletions and duplications, respectively. The green curve shows the Loess regression. Patients 1 to 4 share a q arm deletion. Samples 5 to 7 do not present this deletion. Without defining samples 5 to 7 as controls, only one deletion was correctly detected in patient 4. A partial deletion was detected in patient 1. False-positive deletions were detected in two of the three controls. By defining samples 5 to 7 as controls, two deletions were correctly detected in patients 1 and 4. Two partial deletions were found in both patients 3 and 4. No duplication was found in the controls (partial screenshots from CovCopCan).

We tested this method with the Panel 2 data (Fig 4). Seven samples were simultaneously sequenced on an Ion Proton sequencer (three controls and four patients). The four patients share the same region q deletion on chromosome 13. Without defining controls, CovCopCan detected a correct deletion (highlighted by the vertical orange rectangle) for one of the four patients and only a partial deletion for another. In addition, two false-positive duplications (highlighted by the vertical red rectangle) were detected in two controls. When the control samples were defined (here three controls without the chromosome 13q deletion), CovCopCan efficiently detected two total q deletions on chromosome 13 and two partial deletions for the two other positive patients. In addition, no false-positive duplications were detected in the three controls.

### Performance on germline data

#### Amplicon sensitivity and specificity

We first tested our algorithm on germline data. We used several coverage files obtained after Proton sequencing of our “CMT-89” Ampliseq library (see S1 File, panel 1).

We calculated the sensitivity of CovCopCan, by amplicon, using 22 positive controls confirmed by karyotype, real-time PCR, or Multiplex Ligation-dependent Probe Amplification (MLPA). The detected CNVs were present in 22 patients, sequenced in 11 runs (Table 1). Of the 22 CNVs, 15 are covered by more than 10 amplicons. We used a range of CNV sizes from 4 (TFG) to 98 amplicons (chromosome X duplication). CovCopCan was used with the default settings, with all options active. Raw read values of less than 20 were deleted.

**Table 1:** Details of the 22 positive-control CNVs used for germline analysis, with chromosomal locations of the CNVs. **a**: Number of amplicons covering the CNVs. **b:** Number of amplicons correctly detected as duplicated or deleted by CovCovCan.

Sample	Gene	Chrom.	Start	End	Length (pb)	Amps^a^	Positives Amps^b^	Type
R1_S3	PMP22	chr17	14593353	15167670	574318	10	8	Gain
R1_S8	KIF1A	chr2	241656712	241709233	52522	58	43	Gain
R1_S9	-	chrX	24483480	129299679	104816200	98	94	Gain
R2_S2	AARS	chr16	70286552	70316749	30198	25	22	Gain
R2_S15	DHTKD1	chr10	12110948	12162941	51994	25	25	Loss
R3_S3	KIF1A	chr2	241656712	241709233	52522	58	45	Gain
R4_S4	TFG	chr3	100432328	100439067	6740	4	4	Gain
R4_S12	KIF1A	chr2	241656712	241709233	52522	58	45	Gain
R5_S3	AARS	chr16	70286552	70316749	30198	25	23	Gain
R5_S15	PMP22	chr17	14593353	15167670	574318	10	10	Gain
R5_S16	PMP22	chr17	14593353	15167670	574318	10	10	Loss
R6_S2	PMP22	chr17	14593353	15167670	574318	10	10	Gain
R6_S9	TFG	chr3	100432328	100439067	6740	4	4	Gain
R7_S2	TFG	chr3	100432328	100439067	6740	4	4	Gain
R7_S6	PMP22	chr17	14593353	15167670	574318	10	8	Gain
R8_S8	PMP22	chr17	14593353	15167670	574318	10	10	Loss
R9_S6	PMP22	chr17	14593353	15167670	574318	10	10	Loss
R10_S10	REEP1	chr2	86444070	86509447	65378	7	7	Gain
R10_S16	TFG	chr3	100432328	100439067	6740	4	4	Gain
R11_S8	PMP22	chr17	14593353	15167670	574318	10	10	Gain
R11_S14	TFG	chr3	100432328	100439067	6740	4	3	Gain
R11_S15	REEP1	chr2	86444070	86509447	65378	7	6	Gain

The 22 CNVs are covered by a total of 461 amplicons. CovCopCan correctly detected 403 of 461 deleted/duplicated amplicons, giving an amplicon sensitivity of 0.87. If considering CNV detection, CovCopCan was able to detect 22 of the 22 CNVs tested, leading to a sensitivity of 1.

In addition, we analyzed the *PMP22* gene to calculate the specificity of CovCopCan by amplicon. Indeed, the *PMP22* duplication is the most frequent known mutation responsible for CMT disease and all patients were initially screened by MLPA to detect this gene duplication. The *PMP22* region was covered by 10 amplicons and the entire design contains 2,394 amplicons. We used 456 patients who had no CNV on *PMP22* to estimate the specificity of the CovCopCan algorithm. Of the 4,560 *PMP22* amplicons tested, 4,375 were indeed tagged as “normal” and only 185 were false positives, leading to a specificity of 0.96.

*Comparison with other tools*. We compared CovCopCan with three other tools: IonCopy, DeviCNV, and ExomeDepth. IonCopy and DeviCNV are designed to analyze amplicon sequencing data without a control set. ExomeDepth uses a robust model for the read count data and to build an optimized reference set.

We used the shiny version of the software IonCopy (v. 2.1.1), with the gene-wise analysis mode and default parameters. DeviCNV (v. 1.5.1) was launched with the recommended parameters, detailed in the manual. ExomeDepth (v.0.1) was also launched with the default parameters. We tested these tools on the same dataset, already described, containing the 22 CNVs. We only considered CNVs supported by at least three amplicons for all the tools. The results are presented in Table 2 as the number of CNVs detected.

**Table 2:** Comparison of the performance of CovCopCan and other CNV callers for 22 positive-control CNVs from 22 samples.

	True positives (total = 22)	Other CNVs	Total
**CovCopCan**	22	7	29
**IonCopy**	20	3914	3934
**DeviCNV**	18	117	135
**ExomeDepth**	19	218	237

CovCopCan, IonCopy, DeviCNV, and ExomeDepth each detected 22, 20, 18, and 19 CNVs, respectively (Table 2). Only CovCopCan detected all CNVs for a sensitivity of 1. IonCopy, DeviCNV, and ExomeDepth showed sensitivity of 0.91, 0.82, and 0.86, respectively. It was impossible to verify all the other CNVs found by the various tools. Thus, we could not calculate specificity based on these data. However, a small number of CNVs would be expected, since the data correspond to germline samples. Thus, with only seven CNVs detected in addition to the 22 controls, CovCopCan must have had the best specificity for this dataset.

### Performance on cancer data

#### Low cell fraction

CovCopCan can also process cancer data. The main difference between germline and somatic data is that a cancer tissue sample may simultaneously contain both healthy cells and cancer cells. A low proportion of cancer cells may interfere with the detection of CNVs. We estimated the minimum proportion of cancer cells required for CNV detection by simulating the complete deletion of a gene covered by 80 amplicons using panel 1 (2,394 amplicons). We used a coverage matrix containing the data of 16 patients sequenced by an Ion Proton Sequencer. The deletion of the entire gene was simulated following this method:

SRCi=RRCi×(1−CancerCellProportion)+RRCi2×CancerCellProportion

**Fig 5:**
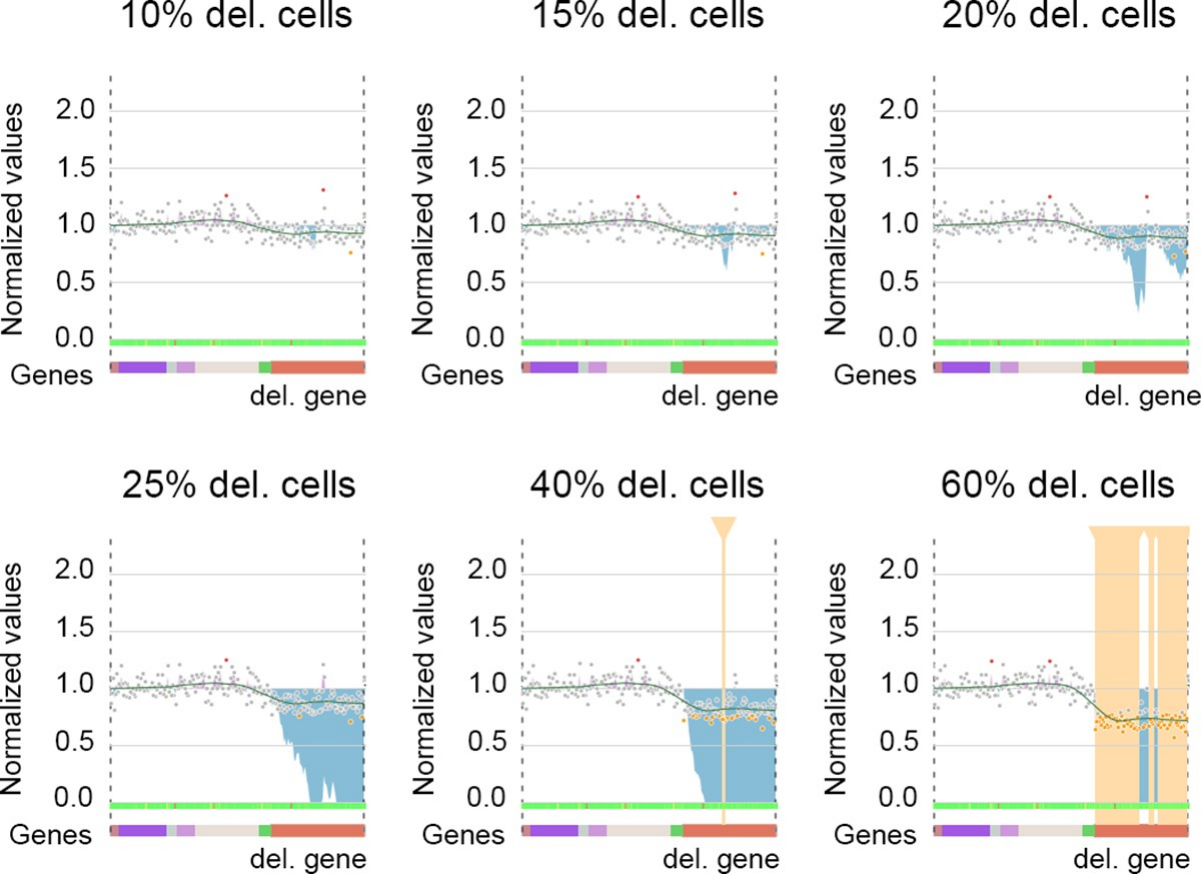
Gene deletion simulation (gene visualized in red), with various proportions of cells containing this deletion. The cumulative summary chart (blue shading) first detected the deletion with 15 to 20% of the cells containing the deletion (partial screenshots from CovCopCan).

*SRC*_*i*_ is the simulated value of the amplicon i, *RRC*_*i*_ the Raw Read Count of the amplicon i, and *CancerCellProportion* the proportion of cancer cells (0 < values < 1). We simulated a proportion of cancer cells ranging from 0 to 1, in steps of 0.05. The first CNV was detected by the cumulative summary chart for 15% of cancer cells and clearly identifiable for 20%. Using only “Z-detection”, the CNV was detected when 40% of the cells contained the deletion, whereas almost the entire gene (67/80 amplicons) was detected by “Z-detection” as deleted for 60% of cancer cells (Fig 5).

**Fig 6:**
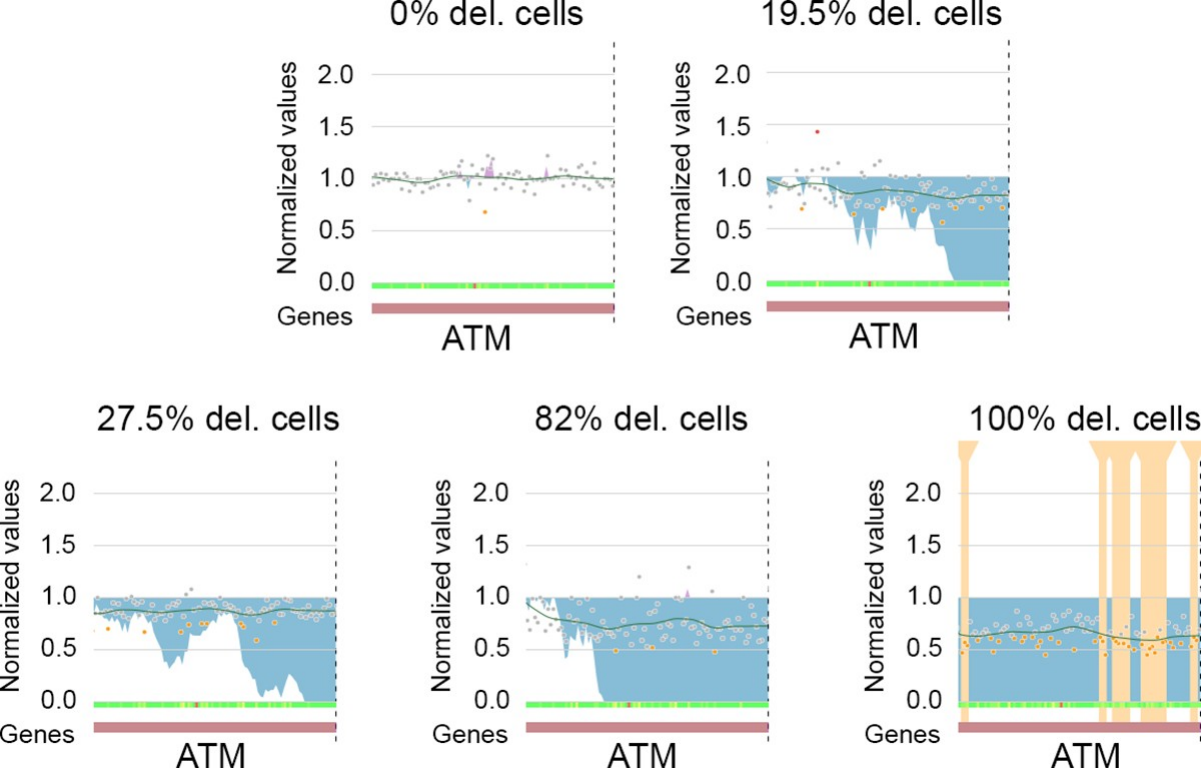
Detection of the entire *ATM* gene deletion in patients DNA, in which the percentage of cancer cells was estimated based on 200 FISH metaphases per patient. The Cumulative summary detected the deletion starting from 19.5% estimated cancer cells (partial screenshots from CovCopCan).

We confirmed the results obtained from these simulated data using real data. We sequenced five patient samples harboring various amounts of positive cancer cells carrying the same *ATM* gene deletion and previously explored with conventional cytogenetics (karyotype and FISH). The data were obtained using panel 2 without control samples. The cumulative algorithm first detected the deletion from 19.5% cancer cells (Fig 6). These results show that CovCopCan can detect CNVs within a heterogeneous sample if the cancer cells make up at least 15 to 20%.

#### Comparison with other tools

We compared the performance of CovCopCan against IonCopy, DeviCNV, and ONCOCNV. First, we used these three tools on the deletion of the *ATM* gene described above. Like CovCopCan, both IonCopy, and ONCOCNV correctly detected the CNV with 19.5% of cancer cells, but not DeviCNV (Table 3).

**Table 3:** Detection of a CNV according to the proportion of cancer cells. “No” indicates no detection of the CNV, whereas “Yes” indicates correct detection of the CNV.

Cancer cell fraction	CovCopCan	IonCopy	DeviCNV	ONCOCNV
0%	No	No	No	No
19.5%	Yes	Yes	No	Yes
27.5%	Yes	Yes	No	Yes
82%	Yes	Yes	Yes	Yes
100%	Yes	Yes	No	Yes

**Fig 7:**
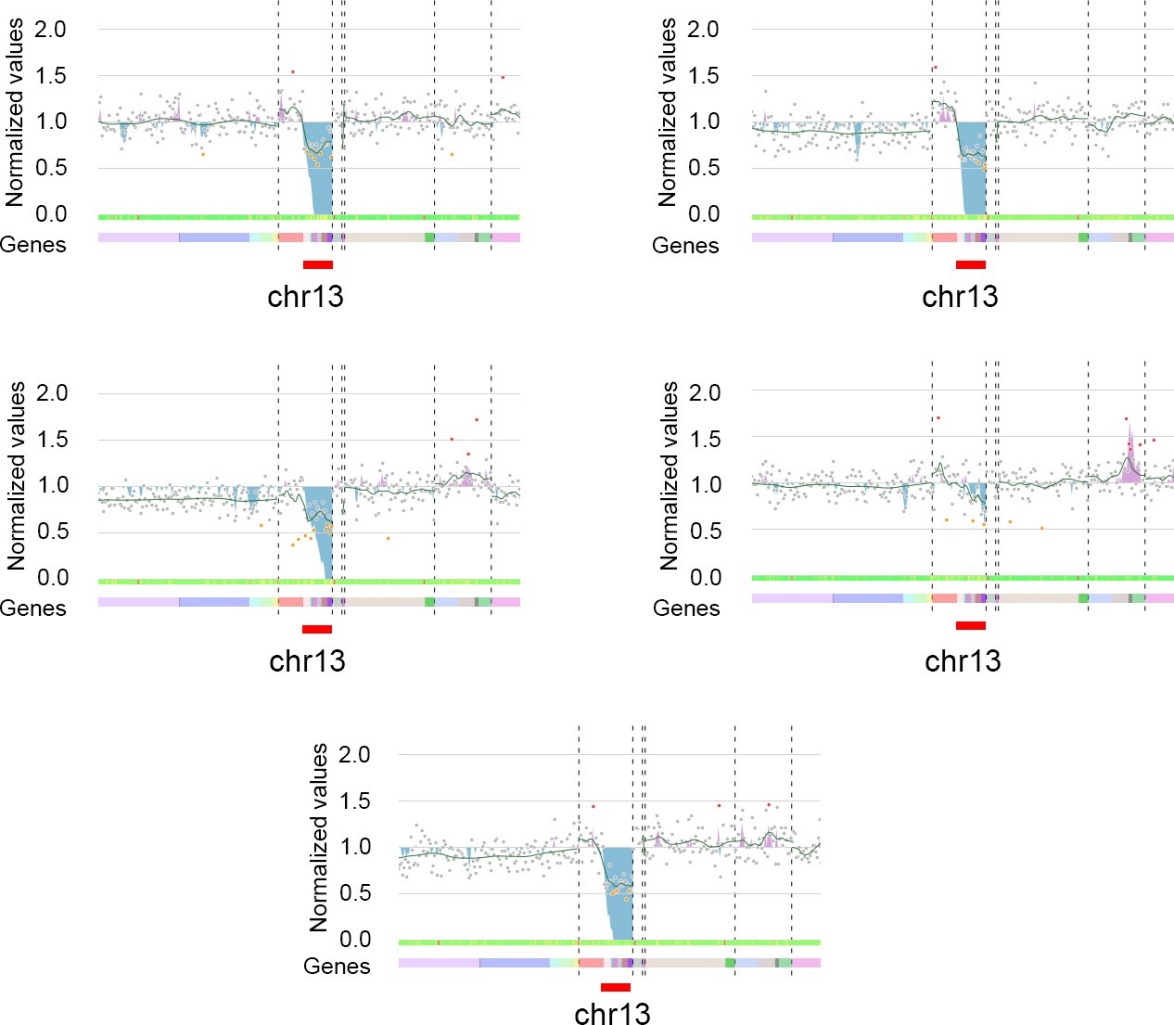
Deletion of the arm of chromosome 13 detected by CovCopCan using the Cumulative Summary Chart. The deletion is highlighted in the blue area.

In addition, we used another dataset obtained using panel 2. We sequenced the DNA of 54 patients in eight runs. Eighteen patients had a partial deletion of a chromosome arm, whereas two had a complete deletion of this same chromosome arm. The partial deletion was covered by 21 amplicons, whereas the entire deletion involved 39. In this study, we did not consider the percentage of cells presenting the CNVs. CovCopCan was used with the default settings, with all options active. Raw read values of less than 20 were deleted. IonCopy was used in the gene-wise mode with the default parameters. DeviCNV was used with the recommended settings. ONCOCNV (v 6.9) was used with the default settings. As with the germline data, we set the minimum number of amplicons to detect CNVs to three for each tool. DeviCNV failed to analyze a run due to a low number of samples (5) and detected four CNVs from the other patients. IonCopy detected nine CNVs. ONCOCNV correctly detected the 20 CNVs but required at least three controls in a run to call them. CovCopCan was able to detect CNVs, with or without controls. Without defining control samples, CovCopCan automatically detected 13 of 20 CNVs. When defining controls, the number of correct CNVs increased to 15 and using the interactive visualization option, such as the CUSUM chart, CovCopCan clearly indicated the presence of a deletion in at least four of the five additional samples (Fig 7).

## Availability and future directions

CovCopCan sources are available on GitHub: https://git.unilim.fr/merilp02/CovCopCan/tree/master. Pre-complied binaries can be downloaded from this page of the GitHub repository: https://git.unilim.fr/merilp02/CovCopCan/tree/master.

CovCopCan offers a wide range of features to interpret data from amplicon sequencing to detect CNVs. This tool works on data generated from Ion Designer (Life Technologies, CA, USA) as well as that from Illumina DesignStudio (Illumina Inc., San Diego, CA, USA). The user-friendly interface associated with our 2D visualization facilitates data exploration and manipulation allowing complex analyses such as those from cancer data. CovCopCan also offers the possibility to export the results in VCF format [16] or graphical output for publications. It can also be used in command-line mode to be integrated into various pipelines (see S1 File).

Future development of CovCopCan will involve the possibility to exploit the variant allele fraction (VAF) to improve the statistical detection of CNVs.

We will also improve memory consumption and parallelism to ensure that CovCopCan can work on a minimal configuration.

## Supporting information

S1 File Supplementary information of this article. The supplementary document provides information on the panels used in this article, a guideline to create an optimized panel to call CNVs, the workflow of CovCopCan algorithm, information on the possibility to define manually reference amplicons, details on graphical visualization elements and command line interface data.
(DOCX)
