# Supplementary material for: CovCopCan: An efficient tool to detect Copy Number Variation from amplicon sequencing data in inherited diseases and cancer
Source: PLoS Comput Biol. 2020 Feb 12;16(2):e1007503. doi: 10.1371/journal.pcbi.1007503 (PMC7041855; doi:10.1371/journal.pcbi.1007503)
Supplement: S1 File — The supplementary document provides information on the panels used in this article, a guideline to create an optimized panel to call CNVs, the workflow of CovCopCan algorithm, information on the possibility to define manually reference amplicons, details on graphical visualization elements and command line interface data. (DOCX) [file pcbi.1007503.s001.docx]

**CovCopCan supplementary data**

# Overall information

CovCopCan is a new tool that allows CNV detection from amplicon sequencing data. The first steps of correction and normalization are identical to those of Cov’Cop: please see details in Derouault *et al*, 2017 [11]. CovCopCan contains a new CNV calling algorithm, based on the z-score, that automatically defines the deletion and duplication thresholds. It can detect deletion or duplication events from cancer data in which the ratio of cancer cells to healthy cells is low, especially if using control samples. Finally, the results are available in an interactive visualization that facilitates their interpretation.

Here, we provide an example using two panels:

**Panel 1**, created using Ion AmpliSeq designer software, includes 89 genes known to be involved in genetic hereditary diseases: Charcot-Marie-Tooth disease (CMT) and closely related pathologies. It contains 2,394 overlapping amplicons and covers 99.26% of the targeted sequences (exons of the genes with a padding of 25 bp), for a total of 585 kb. The average length of the amplicons is 244 bp, ranging from 124 to 274 bp. DNA of 8 to 16 patients is sequenced on an Ion Proton device. The amplification is performed by four multiplex PCR in four distinct tubes, such that two consecutive amplicons for amplification of a gene segment are amplified in two distinct tubes. Libraries were prepared using the Ion AmpliSeq Library kit (v2.0).

**Panel 2**, also created using Ion AmpliSeq designer software, includes 70 genes of interest in cancer. It contains 1,206 amplicons and covers 97.68% of the targeted sequences (exons of the genes with a padding of 10 bp), for a total of 221.6 kb. The average length of the amplicons is 194 bp, ranging from 67 bp to 238 bp. DNA of 7 to 10 patients is sequenced on an Ion Proton device. The amplification is performed by two multiplex PCR in two PCR tubes. Libraries were prepared using the Ion Ampliseq Library kit (v2.0)

# Panel Creation Guide for CNV calling

To create panels that can be used by CovCopCan, we recommend following the recommendations of Ion Ampliseq Designer, particularly for the following points:

- more than 200 amplicons in the panel

- a maximum of 20% of the panel amplicons must be deleted or duplicated

- The CNVs must be covered by at least 10 amplicons. Although we showed that CovCopCan can detect CNVs covered by only four amplicons, increasing the number of amplicons per CNV will reduce the number of false positives. Less than half of the sequenced patients should share the same CNV since CovCopCan compares the sequence of each patient to that of other patients sequenced in the same sequencing run.

CovCopCan can detect large-scale CNVs as aneuploidy (see example of chromosome X duplication in Figure 3 in the main text) and potentially chromothripsis, if the design is adapted to testing the rearranged areas.

# Steps for correction and standardization

## Input files

Two input files are required to run CovCopCan. The first describes the targets of the amplification design. Each line displays information of one amplicon in seven columns: pool, chromosome, start position, end position, amplicon ID, gene name, and sequence.

The second input file is a matrix with the raw read count (RRC) values of each amplicon from each sequenced sample. The first column shows the amplicon ID, the second the gene name linked to the amplicon, and the following columns the RRC for each amplicon (one column corresponding to one patient). We simply create *M_RRC_* from this file, keeping patients in columns and the RRC of the corresponding amplicons in lines:

$$M_{RRC}=\left( \begin{matrix} {RRC}_{A\_1\_P\_1} & \cdots& {RRC}_{A\_1\_P\_m} \\ \vdots& \ddots& \vdots\\ {RRC}_{A\_n\_P\_1} & \cdots& {RRC}_{A\_n\_P\_m} \end{matrix} \right)$$

Thus, in *M_RRC_*, the RRC corresponding to the amplicon *i* belonging to patient *j* is simply denoted *RRC_A_i_P_j_*.

## Correction stage

A succession of corrections is performed, patient by patient, to make them comparable. Multiplex PCR is usually performed using two or more tubes (named pools). Each correction is therefore performed for each pool independently.

**Correction by size of the library**

The first correction step involves scaling of all the RRC values of a sample by the total size of its library. We start the construction of *M_CRC_* as follows:

$${CRC}_{A\_i\_P\_j}=\frac{{RRC}_{A\_i\_P\_j}}{\sum_{i=1}^{n} {RRC}_{A\_i\_P\_j}}$$

${CRC}_{A\_i\_P\_j}$ is the corrected read count of the amplicon $A\_i$ in the sample $P\_j$.

**Correcting the GC-content and the amplicon length bias.**

Amplicons are grouped by their GC rate (increments of 0.1). Then, inside each of these groups, we normalize all *CRC_A_i_P_j_* cells by the group median.

Similarly, the amplicon length bias is corrected in exactly the same way. Amplicons are grouped according to their length of sequences in 10-pb increments.

**Gender correction**

CovCopCan is able to detect female and male samples based on target amplification on the Y chromosome. If there are no targets on the Y chromosome, the user can specify the sex of each sample. Then, all the raw read counts of the amplicons positioned on the X chromosome are multiplied by two for each male sample.

## Normalization/Analysis stages

**Normalization by Reference Amplicons**

After the correction steps, the median and variance of each amplicon among the patients are calculated. Then, reference amplicons are chosen according to two criteria:

- Amplicons exhibiting a minimum variance value
- Amplicons with a median that varies by default in the +/-20% range around the median of the entire matrix.

**Normalization between patients**

These last steps lead to the final matrix *M_CRC_* by giving *CRC_A_i_P_j_* values equal to 1 for a gene present in two copies, to 0.5 for a deletion event, and to 1.5 for a duplication. For each amplicon, each value is divided by the median of the same amplicon in the other samples.

The main steps of the CovCopCan algorithm are presented in Figure A.


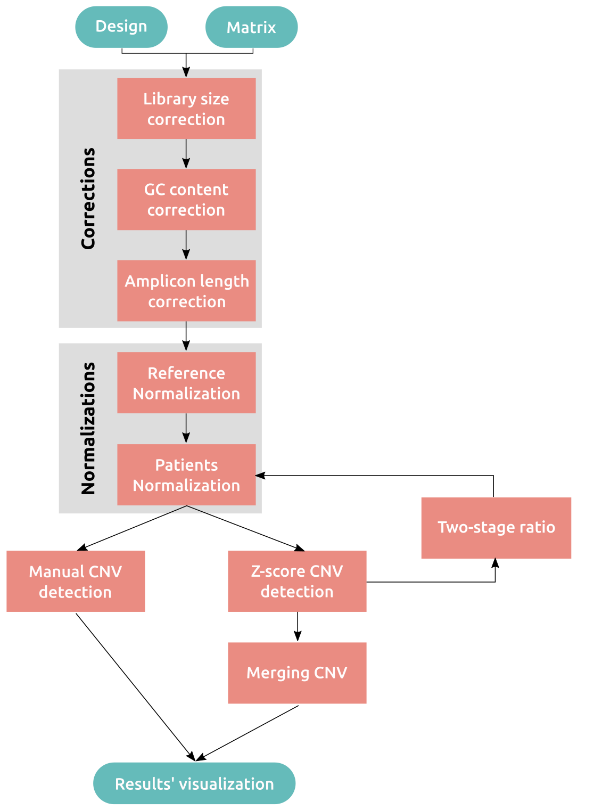


**Figure A.** Workflow of the CovCopCan algorithm.

# Reference

## Reference amplicon

To normalize the data, CovCopCan uses reference amplicons, which are automatically chosen based on their variance and deviation from the average. By default, CovCopCan uses 10% of all amplicons as references. However, users are free to define the percentage of amplicons they want to use as references. In addition, users can also define the reference amplicons to be used by CovCopCan (Figure B).


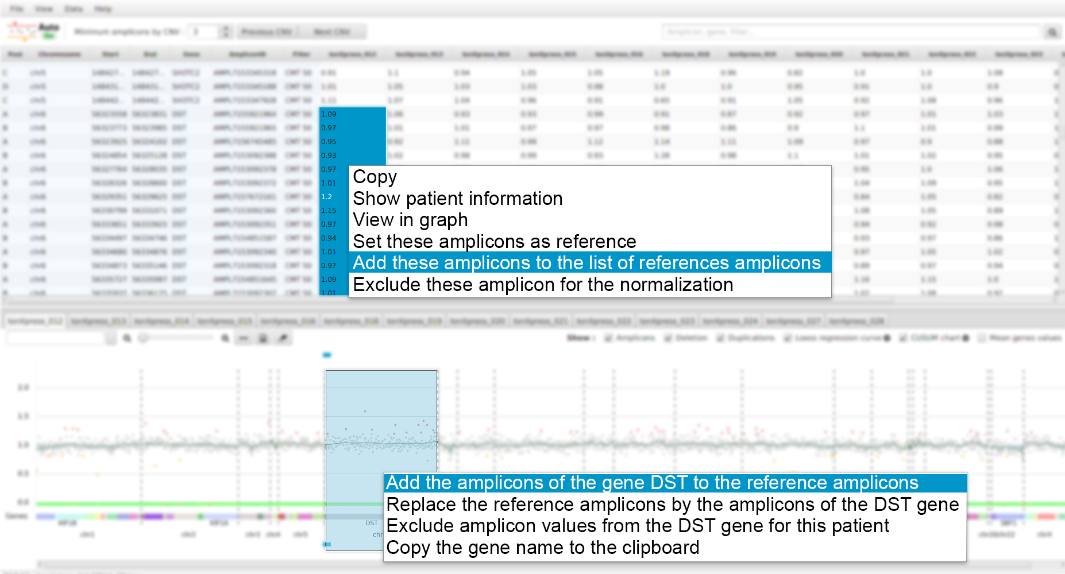


**Figure B.** Reference amplicon option: Reference amplicons can be added from the table (A) or directly from the chart (B).

## Excluded values

Inversely, it is also possible to exclude some amplicon values from the last normalization step. This can be used to manually exclude the amplicons inside a CNV or ignore a disturbed region (Figure C).


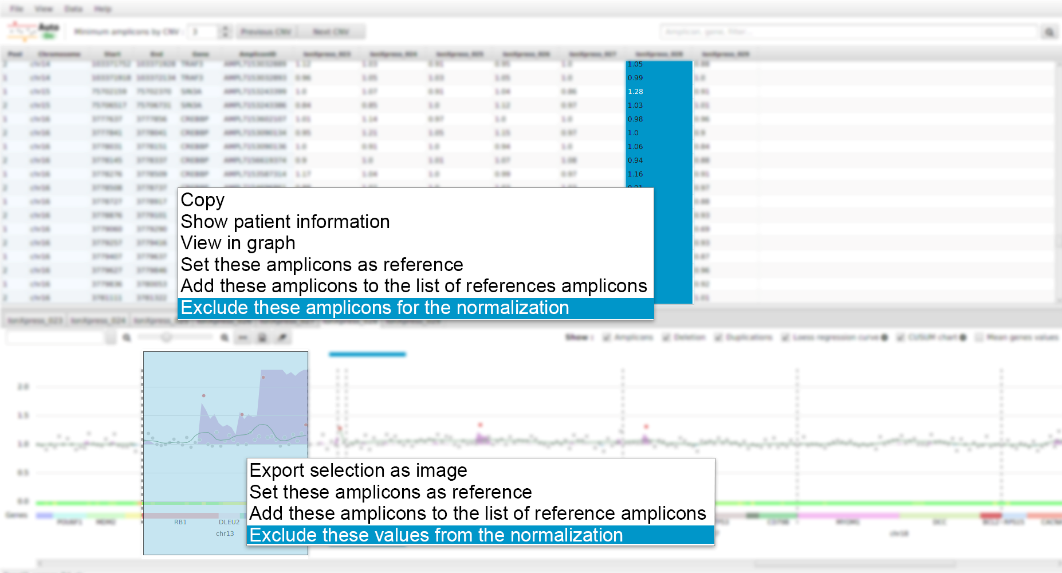


**Figure C.** Excluded amplicon option: User can ask CovCopCan not to use some amplicons as references during the last step of normalization, directly from the table (A) or chart (B).

# Graphical visualization elements

## Loess curve regression

The Loess regression curve can help the user to see small CNVs due to a spike that appears on the Loess curve (Figure D-A). In addition, in the case of cancer or mosaicism, it can help to detect CNVs in a sample containing both cells with CNVs and cells without. The final ratio depends on the proportion of cells with CNVs relative to the others. This curve can be used to highlight a deletion or duplication event characterized by a ratio close to 1 not detected by default as duplicated or deleted by CovCopCan (Figure D-B).


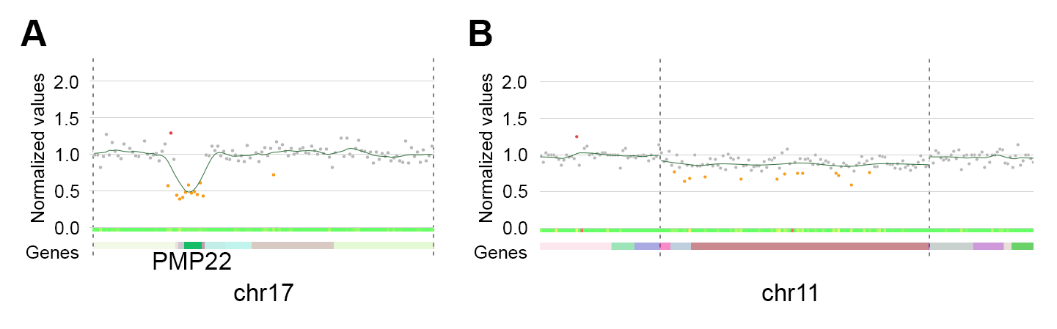


**Figure D.** Loess regression curve. A. Example of the PMP22 deletion: an inverted peak appears on the curve. B. Example of a cancer sample in which all of chromosome 11 is deleted in a small proportion of cells: the Loess curve falls below a value of 1, whereas it is equal to 1 for the surrounding chromosomes. The Loess curve makes such detection of deletions possible.

## Cumulative Summary chart

In addition to the Loess curve, the cumulative summary chart can help to detect deletion and duplication events (not initially highlighted by the vertical orange or red rectangles, respectively). This algorithm calculates the cumulative sum of the positive deviations (values > patient’s average) for deletions and negative deviations (values < patient’s average) for duplications for each chromosome. It can be useful to detect slight deviations of the values, due to low CNV frequency in a subclonal population of cancer cells or germline mosaicism (Figure E-A and B), as well as to detect small CNVs in inherited diseases (Figure E-C and D). Thus, the smaller the deviation, the larger the number of amplicons involved in the CNV needs to be to detect it. Conversely, a few amplicons with a high deviation will be easily highlighted by the CUSUM Charts (Figure E).


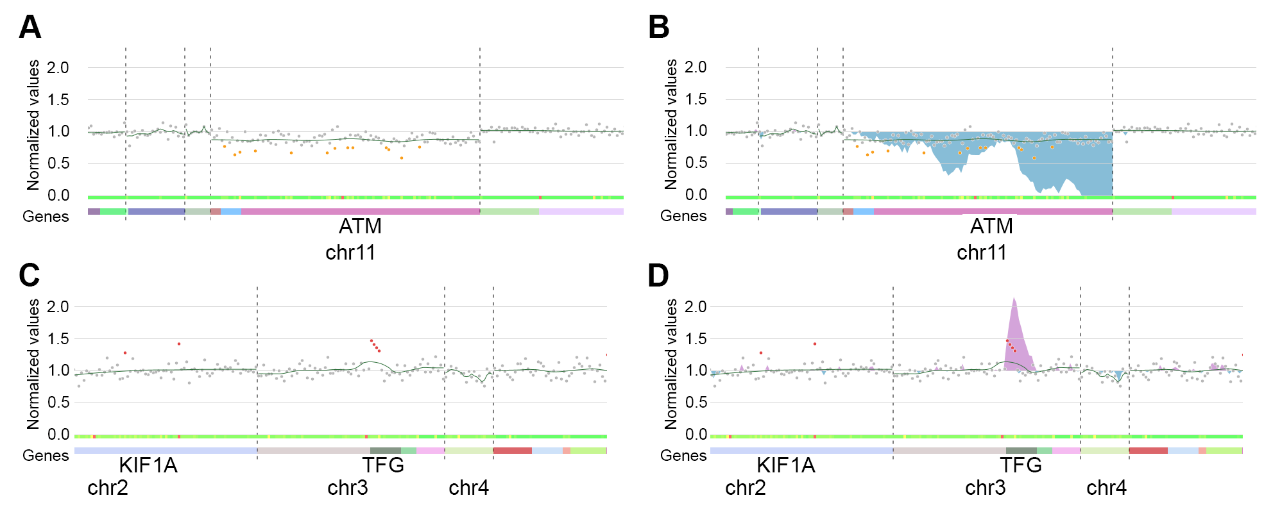


**Figure E.** Cumulative summary control chart. A. Deletion of the chromosome 11, with a ratio close to 1. CovCopCan does not highlight CNVs with vertical orange rectangles because of this ratio value. B. The cumulative summary algorithm indicates the deletion by the blue shading. C. Duplication of a small number of amplicons in gene *TFG*. D. The cumulative summary algorithm helps to identify this duplication by the pink shading.

## Noise heatmap

Each amplicon is associated with a color representing the noise of this amplicon for the different patients of a run. This noise indicates whether the amplicons is stable between the patients or not. We report a score for each amplicon corresponding to the percentage of amplicons tagged as deleted or duplicated between the patients. This score can be estimated by a color scale, in which green indicates a good score and red a bad score. This heatmap can help to visualize a mis-amplified region or a false-positive amplicon.

## Example of the Graphical Visualization of CovCopCan

Figure F illustrates the various elements described above that can be visualized in CovCopCan.


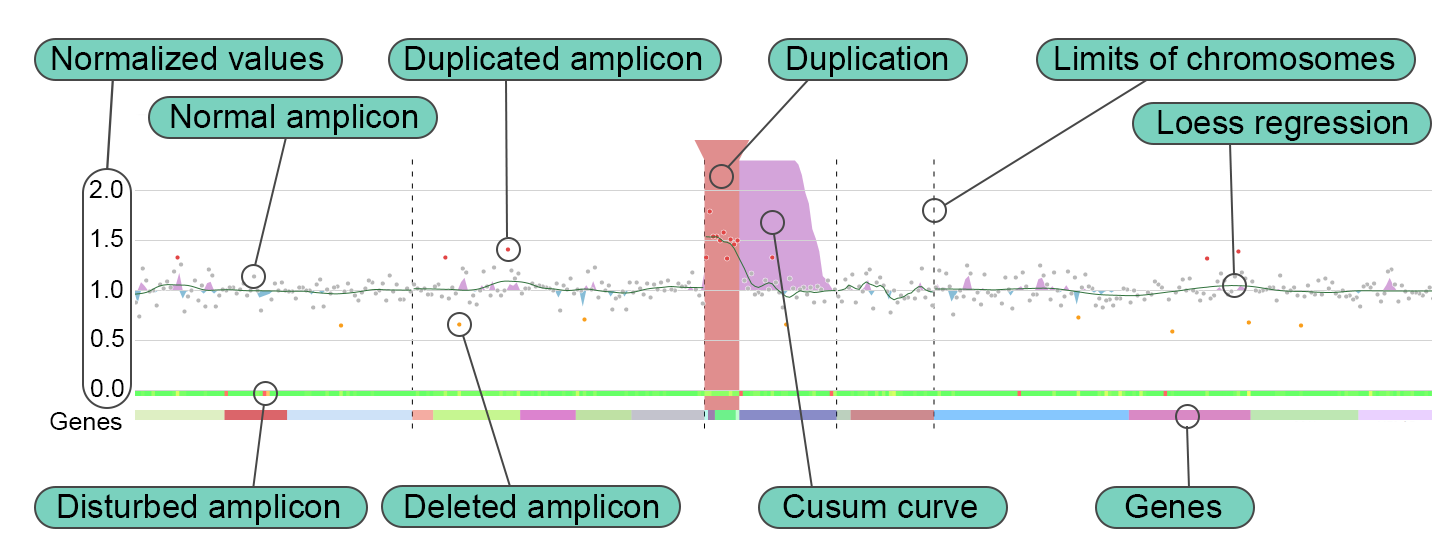


**Figure F.** Representation of all the elements of the CovCopCan visualization.

# Command Line Interface (CLI)

CovCopCan can be run in command line mode to be used automatically and/or can be integrated into an analysis pipeline. The available output are the raw data, the normalized data, the VCF files and the 2D visualization. The CNVs in VCF format follow the recommendations of the Samtools organization (<http://samtools.github.io/hts-specs/>). More precisely, it is formatted according to v.4.3 of the VCF format.

Windows will still open when CovCopCan is used in command-line mode. This is necessary for the program to implement all graphical commands. To use the command-line mode on a Linux server, please use the virtual frame buffer command: xvfb-run. On a Debian system, one can install the virtual frame buffer with the command: apt install xvfb.

Running CovCopCan in command line example on a linux server:
xvfb-run --auto-servernum --server-num=1 java -jar CovCopCan.jar -g -h

CovCopCan has the following command-line options:

-g,--commandLineMode Use CovCopCan in command line mode

-h,--help Show this help message.

-v,--version Print the version of CovCopCan and quit.

-q,--quiet Show only warnings messages.

-d,--design <file> Path to the design file - Required

-m,--matrix <file> Path to the matrix file - Required

-o,--outputDirectory <directory> The output directory to save results - Required

-c,--controlSamples <control1 …> Specify the control samples ID.

--exportRawData <boolean> Export the raw data in TSV file. (Default: true)

--exportNormalizedData <boolean> Export the normalized data in TSV file. (Default: true)

--exportCNVtoVCF <boolean> Export the CNV in VCF files. (Default: true)

--exportChart <boolean> Export the charts. (Default: true)

-r,--cleanMinValue <integer> Remove amplicon below the value. (Default: 20)

--libraryCorrection <boolean> library size correction. (Default: true)

--gcCorrection <boolean> GC content correction. (Default: true)

--ampLenCorrection <boolean> Amplicon length correction. (Default: true)

--twoStepNormalization <boolean> Normalization in two steps. (Default: true)

--referenceAmpliconNumber <integer> Number of amplicons of reference. (Default: 10% of the total amplicons number)

--deviationFromAverage <float> Maximum deviation from average for reference amplicons. (Default: 0.2)

-z,--zScoreDetection <boolean> Use z-score to detect CNV (Default: true)

--deletionThreshold <float> Deletion threshold with no z-score detection. (Default: 0.6)

--duplicationThreshold <float> Duplication threshold with no z-score detection. (Default: 1.4)

--minCNVLength <integer> Minimum number of amplicons to detect CNV (Default: 3)

--showSucum <boolean> Show Sucum chart (Default: false)

--removeSucumOutliers <boolean> Remove the outliers to calculate Sucum chart. (Default: true)

--showAmpliconsDots <boolean> Show amplicons dots. (Default: true)

--showMeanGene <boolean> Show mean gene value (Default: false)

--loessIterate <integer> Loess iterations (Default: 1)

--showLoessCurves <boolean> Show Loess curves (Default: true)

--loessBandwidth <float> Loess bandwidth value (Default: 0.25)

--showDuplications <boolean> Show duplication CNV (Default: true)

--showDeletions <boolean> Show deletions CNV (Default: true)

The -g / --commandLineMode option activates the command line mode of CovCopCan.

The -h/ --help option prints the list of the available commands and their use. Three options are required to run CovCopCan in command-line mode. The first is the path to the design file (-d / --design). The second is the path to the matrix to be analyzed (-m / --matrix). Finally, the path of the output directory is required to save all the results files (-o / --outputDirectory). Then, each feature of the graphical interface is available from the command line mode.

Example of a command to analyze a matrix file without exporting raw data and normalized data and showing the mean average of the gene in the chart:

java -jar CovCopCan.jar -g -d path/to/design/file -m path/to/matrix/file -o path/to/the/outdir –showMeanGene true –exportRawData false –exportNormalizedData false
